# Prenatal Exposure to MAM Impairs mPFC and Hippocampal Inhibitory Function in Mice during Adolescence and Adulthood

**DOI:** 10.1523/ENEURO.0362-24.2024

**Published:** 2024-11-14

**Authors:** Zhiyin He, Qian He, Xiaorong Tang, Keni Huang, Yiwen Lin, Jianrui Xu, Qiliang Chen, Nenggui Xu, Lulu Yao

**Affiliations:** ^1^South China Research Center for Acupuncture and Moxibustion, Medical College of Acu-Moxi and Rehabilitation, Guangzhou University of Chinese Medicine, Guangzhou 510006, China; ^2^School of Basic Medicine, Guangzhou University of Chinese Medicine, Guangzhou 510006, China

**Keywords:** hippocampus, inhibitory function, medial prefrontal cortex, methylazoxymethanol acetate, neuronal activity, parvalbumin, schizophrenia

## Abstract

Neurodevelopmental abnormalities are considered to be one of the important causes of schizophrenia. The offspring of methylazoxymethanol acetate (MAM)–exposed mice are recognized for the dysregulation of neurodevelopment and are well-characterized with schizophrenia-like phenotypes. However, the inhibition-related properties of the medial prefrontal cortex (mPFC) and hippocampus throughout adolescence and adulthood have not been systematically elucidated. In this study, both 10 and 15 mg/kg MAM-exposed mice exhibited schizophrenia-related phenotypes in both adolescence and adulthood, including spontaneous locomotion hyperactivity and deficits in prepulse inhibition. We observed that there was an obvious parvalbumin (PV) loss in the mPFC and hippocampus of MAM-exposed mice, extending from adolescence to adulthood. Moreover, the frequency of spontaneous inhibitory postsynaptic currents (sIPSCs) in pyramidal neurons at mPFC and hippocampus was significantly dampened in the 10 and 15 mg/kg MAM-exposed mice. Furthermore, the firing rate of putative pyramidal neurons in mPFC and hippocampus was increased, while that of putative inhibitory neurons was decreased during both adolescence and adulthood. In conclusion, PV loss in mPFC and hippocampus of MAM-exposed mice may contribute to the impaired inhibitory function leading to the attenuation of inhibition in the brain both in vitro and in vivo.

## Significance Statement

Parvalbumin (PV) neurons could play an important role in maintaining the balance of neural network homeostasis for their ability to provide strong inhibition. PV neurons were reduced in the mPFC and hippocampus in the schizophrenia patients. In this study, we demonstrate that 10 and 15 mg/kg methylazoxymethanol acetate exposure causes a loss of PV neurons in the mPFC and hippocampus of mice, which leads to impaired inhibitory function. Overall, these results provided the significance of PV neurons modulating neural network homeostasis and involvement in the pathogenesis of schizophrenia.

## Introduction

Schizophrenia is a complex psychiatric disorder characterized by positive and negative symptoms, as well as cognitive deficits (Jauhar et al., 2022). There are ∼24 million people or 1 in 300 people worldwide suffering from schizophrenia (World Health Organization, 2020). The pathogenesis of schizophrenia is generally believed to result from a combination of genetic, environmental, neurobiological, and psychological factors that interact in complex ways (McCutcheon et al., 2020a). The current pharmacological treatments of schizophrenia are exclusively dopamine D2 receptor blockers in clinical practice (Kapur and Remington, 2001), which are mainly effective for positive symptoms but have no significant benefit on negative and cognitive symptoms (Leucht et al., 2013; McCutcheon et al., 2020b), even with some adverse effects (Grace and Bunney, 1986; Kapur and Mamo, 2003). Meanwhile, there is increasing evidence showing that excitation–inhibition imbalance in the cortex and hippocampus causes schizophrenia, as it not only leads to negative and cognitive symptoms but also leads to positive symptoms by causing subcortical dopamine overactivity (Calvin and Redish, 2021; Howes and Shatalina, 2022). Therefore, it's important to investigate the pathology of excitation–inhibition of the cortex and hippocampus for the development of therapeutic strategies in schizophrenia.

Common animal models of schizophrenia are established by pharmacological, genetic, or neurodevelopmental disturbance (Białoń and Wąsik, 2022). However, the pharmacological manipulation-induced schizophrenia-like model showed some limitations such as the incomplete manifestation of schizophrenia-like behavior and simulation of an acute rather than a chronic disorder course, while genetic manipulation was time-consuming, expensive, and failed to fully capture the intricacies of schizophrenia (Białoń and Wąsik, 2022). In the study, we adopted the methylazoxymethanol acetate (MAM) administration to the pregnant mice on embryonic day 17 (E17), which corresponds to a critical period of neurogenesis in the rodent brain (Lodge and Grace, 2009). MAM was hypothesized to disrupt neurodevelopment and induce the emergence of schizophrenia symptoms in later life (Lodge and Grace, 2009), including increased locomotor activity, cognitive dysfunction, impaired prepulse inhibition (PPI), and working memory impairment (Lodge and Grace, 2009; Modinos et al., 2015). Thus, we took advantage of this animal model to unravel the schizophrenia-related pathologies at different developmental stages.

The medial prefrontal cortex (mPFC) and hippocampus play a regulatory role in working memory, cognition, emotion, and other aspects, which are considered to be important during the onset of schizophrenia (Harrison, 2004; Xu et al., 2019). The hippocampus is considered to be hyperactive in schizophrenia, which may lead to positive symptoms underlying a hyperresponsive dopamine system, as well as cognitive and negative symptoms underlying dysfunction of the prefrontal cortex and basolateral amygdala–cingulate cortex circuit, respectively (Grace and Gomes, 2019). Both clinical and preclinical evidence showed that the level of parvalbumin (PV) neurons, a marker of fast-spiking GABAergic interneurons, was reduced in the prefrontal cortex and hippocampus in most schizophrenia patients and animal models of schizophrenia, including pharmacological models based on *N*-methyl-d-aspartic acid receptor antagonism, maternal immune activation models, and neuregulin 1 and ERBB4 mutant mice (Konradi et al., 2011; Kaar et al., 2019; Santos-Silva et al., 2024). The decreased density of PV neurons in the hippocampus plays an important role in hippocampal hyperactivity in MAM-exposed (20 mg/kg) rats, manifesting as an increase in the spontaneous firing rate (Lodge and Grace, 2007; Lodge et al., 2009). Investigating the pathology of inhibition in the hippocampus and mPFC will be conducive to reveal the pathogenesis of schizophrenia and find more effective therapeutic targets.

In this study, we performed immunofluorescence, whole-cell patch-clamp recording, and multichannel recording to investigate the expression of PV neurons, its inhibitory regulation of pyramidal neurons in vitro, and the neuronal activity in vivo in mPFC and CA1 in different developmental stages in schizophrenia-like model induced by MAM exposure with different dosage.

## Materials and Methods

### Animals

Male and female C57BL/6J mice of reproductive age were obtained from Guangzhou Zhiyuan Biomedicine Technology. Mice were housed (3–5 mice per cage) in standard conditions (a 12 h light/dark cycle, light on at 7:00) at 23 ± 2°C and 50 ± 5% humidity. Mice had *ad libitum* access to food and water. Clean cages and bedding were replaced at least once a week. All animal procedures were performed in accordance with the Guangzhou University of Chinese Medicine animal care committee's regulations (No. 20230915006) and in accordance with relevant international guidelines and laws and regulations on animal welfare ethics.

### MAM exposure

Two to three female mice cohabited with one male per cage at 18:00 overnight, and the female was checked for vaginal plug at 8:00 the next morning, which with this was recorded as embryonic day 0. Pregnant mice were housed in single cages on E15 and received intraperitoneal MAM (Wako, 136-16303, 10 or 15 mg/kg) or its solvent (0.9% sodium chloride solution, 1 ml/kg) on E17. Pups were housed with their mothers until weaning. The male mice were at adolescence during postnatal days 42–56 (P42–P56) and adulthood during postnatal days 63–77 (P63–P77). The female mice were eliminated.

### Behavioral tests

#### Open-field test

After adapting to the experimental room (1 h), mice were placed gently in the central area of the open field (40 × 40 × 40 cm) and allowed to move freely for 30 min. After each round, the arena was cleaned and sprayed with 20% alcohol to eliminate odors left by the mice. Finally, the total distance and central times of the mice in the open field were analyzed (Jiliang Software Technology).

#### Three-chamber social test

After adapting to the experimental room (1 h), mice were tested for social interaction in a blue plexiglass rectangular box (60 × 40 × 30 cm) consisting of three interconnected chambers. This experiment was divided into three sessions: in Session 1, mice were allowed to freely explore the box for 5 min to acclimate to the three interconnected chambers; in Session 2, a plexiglass cylinder was placed in the lower left corner of the left chamber and in the upper right corner of the right chamber to serve as the “nonsocial” and “social” cylinders for Session 3, respectively. Mice explored freely for 10 min to acclimate to the two new cylinders; in Section 3, a stranger mouse was placed inside the “social” cylinder, and the test mice were allowed to explore for another 10 min. Time spent around each cylinder was recorded and analyzed using a tracking system (Jiliang Software Technology). After each section, the box or cylinder was cleaned and sprayed with 20% alcohol to eliminate odors.

#### Y-maze test

Three arms (25 × 10 × 25 cm) of the Y maze were labeled as Arm A, Arm B, and Arm C, respectively. After acclimated to the experimental room (1 h), mice were placed in the same position of the Y maze (the end of Arm A) and allowed to freely traverse the three arms for 8 min, which was recorded by a tracking system (Jiliang Software Technology). When the mice entered three different arms in succession, it was recorded as an alternation. The apparatus was cleaned after each round and sprayed with 20% alcohol to eliminate odors. The formula for the percentage of spontaneous alternation behavior is as follows: Alternation (%) = 100 × (number of alternation / (total number of arm intake − 2)).

#### Prepulse inhibition test

PPI test was performed using the SR-LAB System device (San Diego Instruments), according to the manufacturer's protocol. In the upfront phase, mice were acclimated to the chambered for 5 min in 70 dB background white noise, followed by the onset of formal pseudorandom stimulation. Mice received 12 startle trials (20 ms, 120 dB) and 12 prepulse/startle trials (20 ms white noise of 75, 80, or 85 dB at 100 ms intervals and 120 dB startle stimulus). After each test, the chamber was cleaned and sprayed with 20% ethanol. PPI (%) = 100 × (startle amplitude of individual pulses − startle amplitude for the pulse with prepulse) / startle amplitude of individual pulses.

### Immunofluorescence

Mice were anesthetized by intraperitoneal injection of 1.25% avertin and then perfused with 0.9% sodium chloride solution followed by 4% PFA. The mouse brain was removed and postfixed by immersion in 4% PFA overnight and then dehydrated sequentially in 15% (12 h) and 30% (above 24 h) sucrose solutions at 4°C. Coated with an optimal cutting temperature compound, the brain was cut coronally into 40 µm slices with a freezing microtome (Thermo Fisher Scientific). After washing with 0.01 M PBS (3 × 8 min), slices were blocked in a buffer solution containing 2% BSA (B885114, Macklin), 10% goat serum (AR0009, Boster), and 0.5% Triton X-100 (BL935A, Biosharp) at 37°C for 1 h and then incubated with primary antibodies (rabbit anti-parvalbumin, 1:1,000, ab11427, Abcam) at 4°C for 24 h, followed by Alexa Fluor 488–conjugated goat anti-rabbit secondary antibody (1:500, ab150077, Abcam) at 37°C for 1 h. After labeling the nuclei with DAPI (1 µg/5 ml, D9542, Sigma-Aldrich) for 5 min, slices were mounted on slides and sealed. The images were obtained at 20× on a confocal microscope (Nikon Eclipse Ti) including two laser lines (405 and 488 nm), at a step size of 2 µm for 20 μm thickness of the tissue slice. The total number of PV^+^ cells in dorsal CA1 (dCA1), ventral CA1 (vCA1), and mPFC (2–5 slices per mouse) were obtained using ImageJ (NIH).

### Whole-cell patch-clamp recordings

Anesthetized by intraperitoneal injection of 1.25% avertin, the mouse brain was rapidly removed and soaked in the ice-cold cutting solution containing the following (in mM): 2.5 KCl, 1.25 NaH_2_PO_4_-2H_2_O, 25 NaHCO_3_, 25 d-glucose, 110 choline chloride, 0.5 CaCl_2_-2H_2_O, 7 MgSO_4_, 11.6 Na-ascorbate, and 3.1 Na-pyruvate, bubbled with 95% O_2_ and 5% CO_2_. mPFC or hippocampus coronal slices (300 µm) were acquired with a vibrating microtome (VT 1200S, Leica). The slices were placed in artificial cerebrospinal fluid (ACSF) containing the following (in mM): 127 NaCl, 2.5 KCl, 1.25 NaH_2_PO_4_-2H_2_O, 25 NaHCO_3_, 25 d-glucose, 2 CaCl_2_-2H_2_O, and 1 MgCl_2_, bubbled with 95% O_2_ and 5% CO_2_ and incubated at 34°C for 30 min, followed by 1 h at room temperature.

The slices were then transferred to a chamber perfused with ACSF bubbled with 95% O_2_ and 5% CO_2_ at 3 ml/min. Pyramidal neurons in the mPFC (layers 5–6) and CA1 were identified based on their large soma size and long apical dendrite (Zhang et al., 2021) under the infrared-sensitive CCD camera (ORCA-Flash4.0 LT C11440, Hamamatsu Photonics) with a 40× water immersion lens (NIR APO DIC N2, Nikon). Whole-cell voltage-clamp recording (holding at +10 mV) was used to acquire spontaneous inhibitory postsynaptic currents (sIPSCs) at room temperature. Patch pipettes (4–7 MΩ) were pulled out of borosilicate capillary glass (BF150-86-10, Sutter Instrument) using a flaming/brown micropipette puller (P-97, Sutter Instrument), in which the solution contained the following (in mM): 125 CsCH_3_SO_3_, 10 HEPES, 5 CsCl, 0.2 EGTA, 4 Mg-ATP, 1 MgCl_2_, 5 QX-314, 10 phosphocreatine, and 0.3 Na-GTP (pH 7.30, 280–300 mOsm). When series resistance fluctuated within 20% of initial values (10–20 MΩ), data were collected using a MultiClamp 700B amplifier and 1550B digitizer (Molecular Devices), filtered at 1 kHz, and sampled at 10 kHz, which later offline processed with Clampfit 10.7 (Molecular Devices) and analyzed with MiniAnalysis (Synaptosoft).

### In vivo electrophysiology

The 35-d-old mice were anesthetized with 1–2% isoflurane in an oxygen/air mixture and then immobilized on a brain stereotactic apparatus (RWD Life Science). The scalp was cut in the middle to expose the skull, and then a small hole (3 × 3 mm) was polished on the skull face using a skull drill. After that, a nichrome electrode was implanted into mPFC (AP, 0.37 mm; ML, 2 mm; DV, −2.5 mm) or vCA1 (AP, −3.2 mm; ML, 3.28 mm; DV, −4.4 mm), which was fixed with inserting three screws and dental cement. The mice were recovered for 7–14 d after surgery. In vivo recordings of mice in adolescence were performed at 42–56 d old and those in adulthood at 63–77 d old. Spike signals were collected with the OmniPlex Neural Recording Data Acquisition System (16 bits, 30 kHz, Plexon) for 5 min and analyzed with Offline Sorter software (Plexon). The units were divided into clusters of neurons with similar firing properties.

Neurons were divided into putative pyramidal neurons and putative inhibitory neurons based on the waveform and firing rate of spikes as follows. (1) Peak–peak spacing: pyramidal neurons dominate at short peak–peak spacing (3–10 ms), with a distinctive exponential decline post a signature 3–5 ms interval, contrasting the longer spike latency and slower decay observed in inhibitory neurons. (2) Spike asymmetry: compared to the peak value after the valley point, pyramidal cells exhibit a larger peak value before the valley point, a pattern inverse to that of inhibitory neurons where the peak value after the valley point is larger. (3) Peak-to-peak ratio: pyramidal neurons generally have a gentle waveform with smaller peaks and smaller interpeak values. After recording, the mice were killed, and brain slices were used to confirm the recording site.

### Nissl staining

Brain slices were obtained by the same method as for immunofluorescence. The brain slices were spread on attached glass slides and soaked in 70% ethanol for 4 h, followed by 2 min in double distilled water. Subsequently, the brain slices were stained with Nissl staining solution (Beyotime), which was carried out in a constant temperature incubator at 50°C for 15 min. After staining, brain slices were dehydrated with absolute ethanol (2 × 5 min) and then transparent with xylene (5 min) and finally sealed with neutral gum.

### Data analysis and statistical tests

GraphPad Prism 10 software was used for statistical analysis and statistical mapping. Data were tested for normal distribution with the Shapiro–Wilk test and homogeneity of variance with the Brown–Forsythe test. Data with normal distribution and homogeneity of variance were analyzed by one-way ANOVA or two-way ANOVA with Tukey's post hoc test, while non-normally distributed data were analyzed by Kruskal–Wallis with Dunn's post hoc test. All data are reported as the mean ± SEM. All tests were two-sided, and *p *< 0.05 was considered to indicate statistical significance.

## Results

### A loss of PV neurons in mPFC and CA1 during adolescence and adulthood

It was well-known that the offspring of rodents injected with 20–26 mg/kg MAM on E17 exhibited schizophrenia-like behaviors (Ruda-Kucerova et al., 2017; Gill et al., 2018; Zhang et al., 2023; Péczely et al., 2024). However, we found that intraperitoneal injection of 20 mg/kg MAM on E17 in mice resulted in the death of the pups due to maternal aggression or failure to suckle the pups in the study. Therefore, we have selected two lower doses of MAM (10 or 15 mg/kg) to ensure the survival and emergence of schizophrenia-like symptoms as possible. Mice exposed to low-dose MAM conceived and gave birth successfully, and most pups survived and appeared healthy. Furthermore, the results showed that 10 or 15 mg/kg MAM-exposed mice exhibited schizophrenia-like behaviors in both adolescence and adulthood, as revealed in locomotor hyperactivity in the open-field test and a lower level of PPI in prepulse inhibition test (Extended Data Figs. 1-1, 1-2).

**Figure 1.**
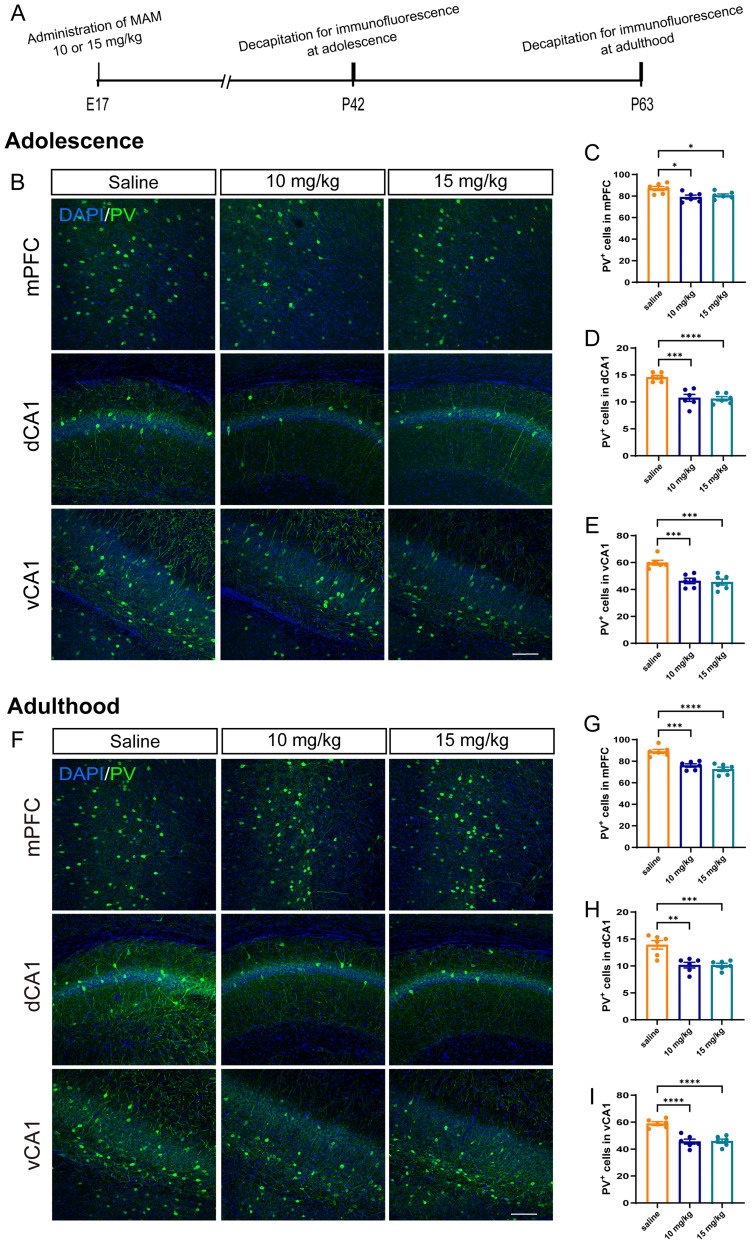
Decreased quantity of parvalbumin-immunoreactive neurons in mPFC and CA1 during adolescence and adulthood in MAM-exposed mice. ***A***, Schematic diagram of the immunofluorescence experimental procedure for staining PV neurons. ***B***, Representative images of PV neurons in the mPFC, dCA1, and vCA1 of saline- and MAM (10 and 15 mg/kg)-exposed mice during adolescence. Scale bar, 100 μm. ***C–E***, Number of PV neurons in the mPFC (***C***), dCA1 (***D***), and vCA1 (***E***) of saline- and MAM (10 and 15 mg/kg)-exposed mice during adolescence, respectively. ***F***, Representative images of PV neurons in the mPFC, dCA1, and vCA1 of saline- and MAM (10 and 15 mg/kg)-exposed mice during adulthood. Scale bar, 100 μm. ***G–I***, Number of PV neurons in the mPFC (***G***), dCA1 (***H***), and vCA1 (***I***) of saline- and MAM (10 and 15 mg/kg)-exposed mice during adulthood, respectively. *N *= 6 mice per group. Data are mean ± SEM. *p < 0.05, **p < 0.01, ***p < 0.001, ****p < 0.0001. Figure contributions: Z.H. and Q.H. performed the experiments and analyzed the data; Y.L. and J.X. assisted in the data analysis. See Extended Data Figures 1-1 and 1-2 for more details.

The PV neurons in the cortex and hippocampus are closely associated with schizophrenia (Lewis et al., 2005; Gill and Grace, 2014; Du and Grace, 2016). Besides, a systematic review and meta-analysis showed that CA1 was the most affected region among the various hippocampal subregions in schizophrenia (Haukvik et al., 2018), so we focused on the dCA1 and vCA1. We first detected the expression of PV in the mPFC and CA1 (Fig. 1*A,B,F*). The immunofluorescent staining result showed that the number of PV neurons in the both 10 and 15 mg/kg MAM-exposed mice exhibited a reduction in the mPFC (*F*_(2,15) _= 6.124, *p *= 0.0114; Fig. 1*C*), dCA1 (*F*_(2,15) _= 22.56, *p *< 0.0001; Fig. 1*D*), and vCA1 (*F*_(2,15) _= 16.04, *p *= 0.0002; Fig. 1*E*) during adolescence, and this alteration persisted into adulthood (mPFC, *F*_(2,15) _= 23.17, *p *< 0.001, Fig. 1*G*; dCA1, *F*_(2,15) _= 13.92, *p *= 0.0004, Fig. 1*H*; and vCA1, *F*_(2,15) _= 23.90, *p *< 0.0001, Fig. 1*I*). These results suggested that the administration of 10 or 15 mg/kg MAM led to PV loss in mPFC and CA1 in male mice, from adolescence to adulthood.

10.1523/ENEURO.0362-24.2024.f1-1 Figure 1-1 **10  mg/kg and 15  mg/kg MAM-exposed mice exhibited schizophrenia-like behaviors during adolescence. *A***, Schematic representation of Saline- and MAM (10  mg/kg and 15  mg/kg)- exposed mice undergoing a series of schizophrenia-related behavioral tests during adolescence. ***B-D***, MAM exposure induced locomotor hyperactivity in the open field test. Representative activity tracking (B), total distance (C) and time in center (D) [(C) *F*(2,22) = 7.616, *p* = 0.0031; (D) *F*(2,22) = 0.852, *p* = 0.4400]. ***E***, ***F***, No changes in social activity were observed. Representative activity tracking (E) and social preference indexes (F) in three-chambers social test [*F*(2,22) = 3.207, *p* = 0.0599]. ***G***, No changes in alternation (%) in Y maze test [*F*(2,22) = 0.953, *p* = 0.4011]. ***H***, ***I***, MAM exposure induced an impaired PPI. Quantification of response to 70  dB (E) and percentage of PPI (F) in prepulse inhibition test [(E) *p* = 0.2761; (F) Two-way ANOVA, interaction: *F*(4, 66) = 0.323, *p* = 0.8619; main effect of decibel: *F*(2, 66) = 11.230, *p* < 0.0001; main effect of group: *F*(2, 66) = 19.120, *p* < 0.0001]. *N* = 8, 9, 8 mice per group. Data are mean ± SEM. *p < 0.05, **p < 0.01, ***p < 0.001. *Figure Contributions*: Zhiyin He and Keni Huang performed the experiments and analyzed the data. Download Figure 1-1, TIF file.

**Figure 2.**
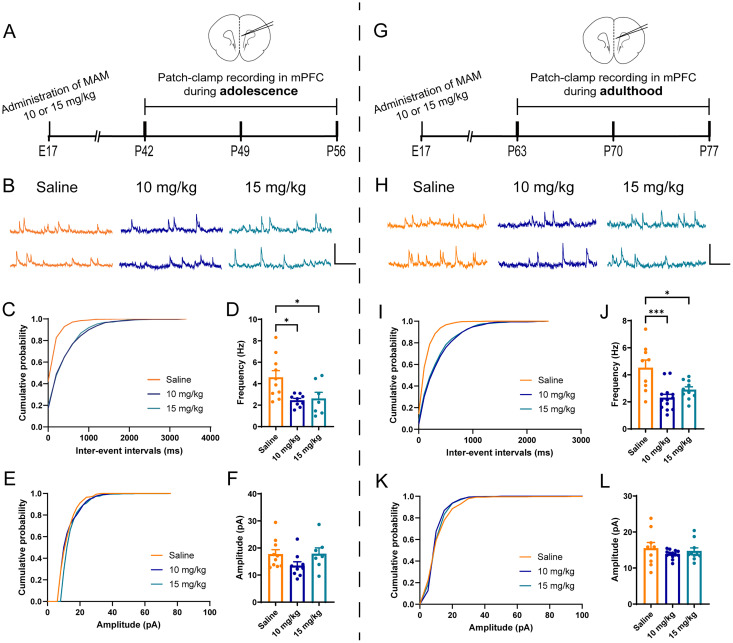
Impaired inhibition in mPFC of MAM-exposed mice. ***A***, Schematic diagram of whole-cell patch-clamp recording in mPFC during adolescence. ***B***, Representative traces of sIPSCs in mPFC pyramidal neurons from saline- and MAM (10 and 15 mg/kg)-exposed mice during adolescence. Calibration, 400 ms, 20 pA. ***C***, ***D***, Cumulative distribution of sIPSC interevent intervals (***C***) and average frequencies (***D***). ***E***, ***F***, Cumulative distribution of sIPSC amplitudes (***E***) and average amplitudes (***F***). *N *= 10, 9, and 7 cells from 3 to 4 mice per group, respectively. ***G***, Schematic diagram of whole-cell patch-clamp recording in the mPFC during adulthood. ***H***, Representative traces of sIPSCs in mPFC pyramidal neurons from saline- and MAM (10 and 15 mg/kg)-exposed mice during adulthood. Calibration, 400 ms, 20 pA. ***I***, ***J***, Cumulative distribution of sIPSC interevent intervals (***I***) and average frequencies (***J***). ***K***, ***L***, Cumulative distribution of sIPSC amplitudes (***K***) and average amplitudes (***L***). *N *= 9, 13, and 10 cells from 3 mice per group, respectively. Data are mean ± SEM. *p < 0.05, ***p < 0.001. Figure contributions: Q.H. performed the experiments and analyzed the data.

10.1523/ENEURO.0362-24.2024.f1-2 Figure 1-2 **10  mg/kg and 15  mg/kg MAM-exposed mice exhibited schizophrenia-like behaviors during adulthood. *A***, Schematic representation of Saline- and MAM (10  mg/kg and 15  mg/kg)- exposed mice undergoing a series of schizophrenia-related behavioral tests during adolescence. ***B-D***, MAM exposure induced locomotor hyperactivity in the open field test. Representative activity tracking (B), total distance (C) and time in center (D) [(C) *F*(2,25) = 6.897, *p* = 0.0041; (D) *F*(2,25) = 0.122, *p* = 0.8858]. ***E***, ***F***, No changes in social activity were observed. Representative activity tracking (E) and social preference indexes (F) in three-chambers social test [*F*(2,25) = 0.200, *p* = 0.8201]. ***G***, No changes in alternation (%) in Y maze test [*F*(2,25) = 0.394, *p* = 0.6787]. ***H***, ***I***, MAM exposure induced an impaired PPI. Quantification of response to 70  dB (E) and percentage of PPI (F) in prepulse inhibition test [(E) *p* = 0.7745; (F) Two-way ANOVA, interaction: *F*(4, 75) = 0.575, *p* = 0.6820; main effect of decibel: *F*(2, 75) = 64.010, *p* < 0.0001; main effect of group: *F*(2, 75) = 18.370, *p* < 0.0001]. *N* = 10, 9, 9 mice per group. Data are mean ± SEM. *p < 0.05, **p < 0.01, ***p < 0.001. *Figure Contributions*: Zhiyin He and Qian He performed the experiments and analyzed the data. Download Figure 1-2, TIF file.

### A reduction of sIPSCs in pyramidal neurons at mPFC and CA1 in vitro

**Figure 3.**
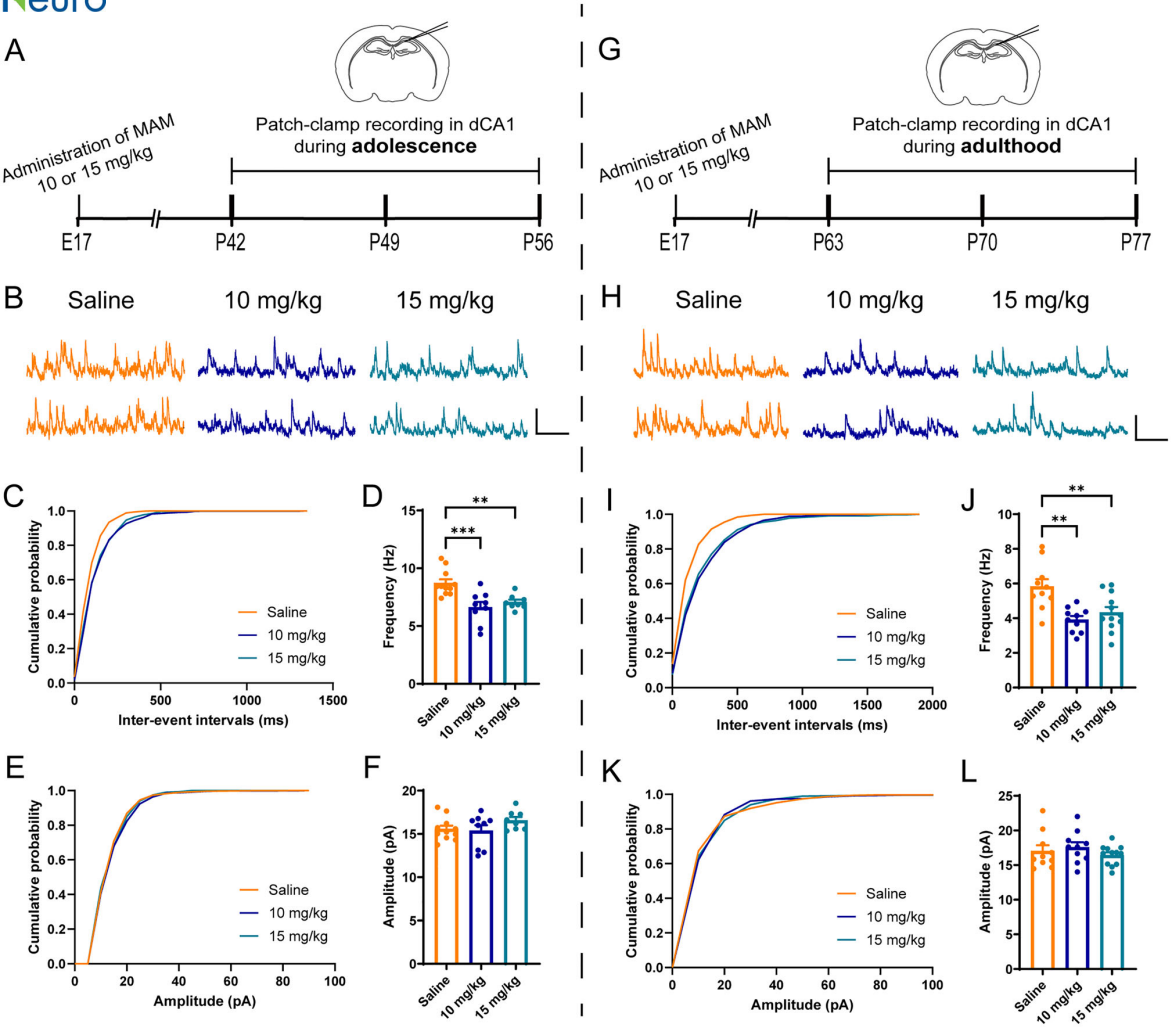
Impaired inhibition in dCA1 of MAM-exposed mice. ***A***, Schematic diagram of whole-cell patch-clamp recording in dCA1 during adolescence. ***B***, Representative traces of sIPSCs in dCA1 pyramidal neurons from saline- and MAM (10 and 15 mg/kg)-exposed mice during adolescence. Calibration, 400 ms, 20 pA. ***C***, ***D***, Cumulative distribution of sIPSC interevent intervals (***C***) and average frequencies (***D***). ***E***, ***F***, Cumulative distribution of sIPSC amplitudes (***E***) and average amplitudes (***F***). *N *= 11, 9, and 8 cells from 3 mice per group, respectively. ***G***, Schematic diagram of whole-cell patch-clamp recording in dCA1 during adulthood. ***H***, Representative traces of sIPSCs in dCA1 pyramidal neurons from saline- and MAM (10 and 15 mg/kg)-exposed mice during adulthood. Calibration, 400 ms, 20 pA. ***I***, ***J***, Cumulative distribution of sIPSC interevent intervals (***I***) and average frequencies (***J***). ***K***, ***L***, Cumulative distribution of sIPSC amplitudes (***K***) and average amplitudes (***L***). *N *= 10, 10, and 12 cells from 3 to 4 mice per group, respectively. Data are mean ± SEM. **p < 0.01, ***p < 0.001. Figure contributions: Z.H. performed the experiments and analyzed the data.

**Figure 4.**
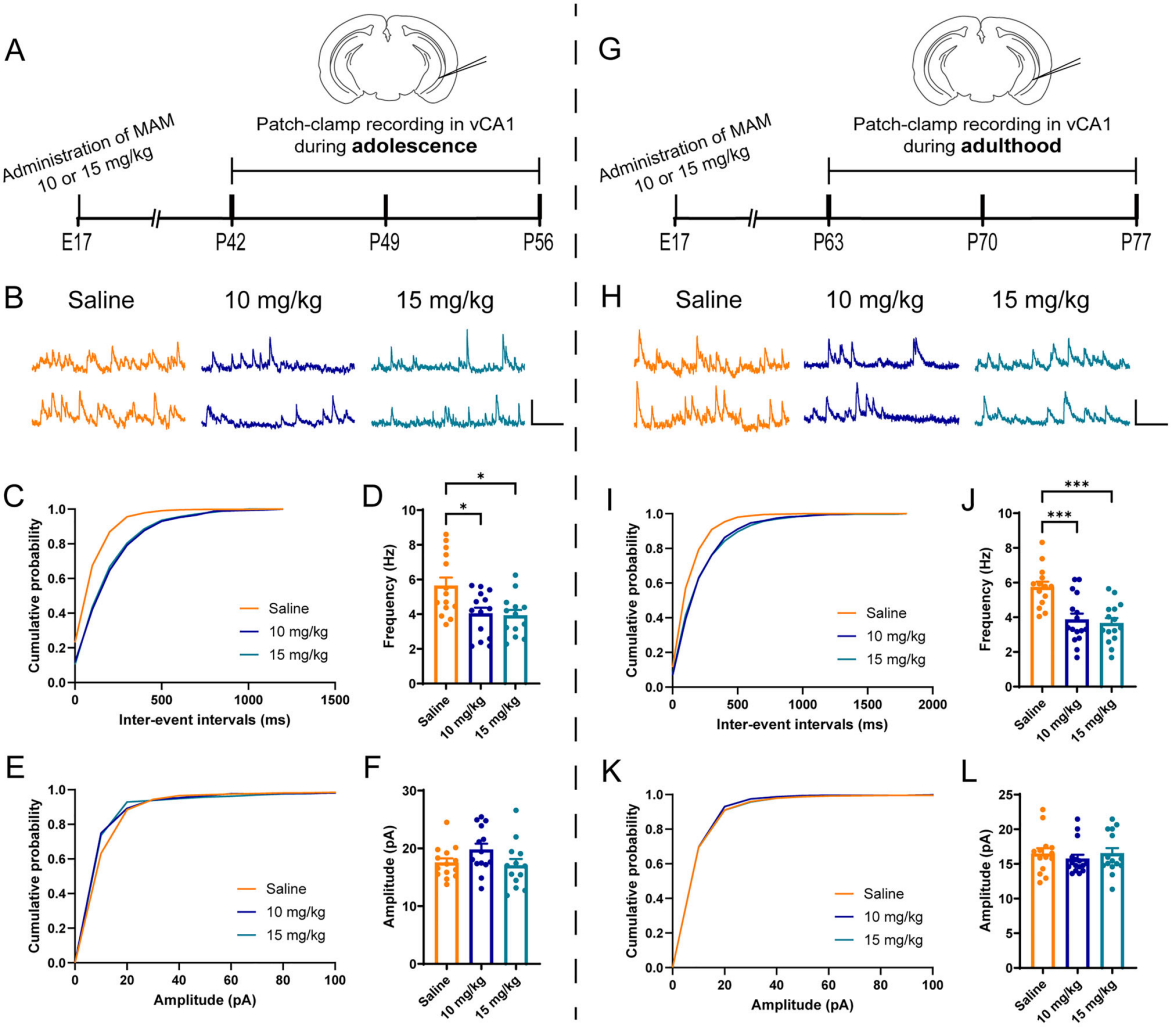
Impaired inhibition in vCA1 of MAM-exposed mice. ***A***, Schematic diagram of whole-cell patch-clamp recording in vCA1 during adolescence. ***B***, Representative traces of sIPSCs in vCA1 pyramidal neurons from saline- and MAM (10 and 15 mg/kg)-exposed mice during adolescence. Calibration, 400 ms, 20 pA. ***C***, ***D***, Cumulative distribution of sIPSC interevent intervals (***C***) and average frequencies (***D***). ***E***, ***F***, Cumulative distribution of sIPSC amplitudes (***E***) and average amplitudes (***F***). *N *= 14, 14, and 13 cells from 4 mice per group, respectively. ***G***, Schematic diagram of whole-cell patch-clamp recording in vCA1 during adulthood. ***H***, Representative traces of sIPSCs in vCA1 pyramidal neurons from saline- and MAM (10 and 15 mg/kg)-exposed mice during adulthood. Calibration, 400 ms, 20 pA. ***I***, ***J***, Cumulative distribution of sIPSC interevent intervals (***I***) and average frequencies (***J***). ***K***, ***L***, Cumulative distribution of sIPSC amplitudes (***K***) and average amplitudes (***L***). *N *= 14, 16, and 15 cells from 3 to 5 mice per group, respectively. Data are mean ± SEM. *p < 0.05, ***p < 0.001. Figure contributions: Z.H. performed the experiments and analyzed the data.

To validate whether PV loss in mPFC and CA1 in 10 and 15 mg/kg MAM-exposed mice could affect the inhibition toward the pyramidal neurons, we used patch-clamp whole-cell recordings, to measure sIPSCs in pyramidal neurons in mPFC, dCA1, and vCA1. In mPFC (Fig. 2*A,G*), the results showed a reduction in the frequency of sIPSCs (*F*_(2,23) _= 5.849, *p *= 0.0088; Fig. 2*B–D*) but no alternation in the amplitude (*F*_(2,23) _= 1.982, *p *= 0.1606; Fig. 2*B,E,F*) during adolescence in either 10 mg/kg MAM-exposed mice or 15 mg/kg MAM-exposed mice. Furthermore, the frequency of sIPSCs in mPFC decreased in both 10 mg/kg MAM-exposed mice and 15 mg/kg MAM-exposed mice in adulthood (*F*_(2,29) _= 10.05, *p *= 0.0005; Fig. 2*H–J*), while the amplitude showed no alteration (*F*_(2,29) _= 0.7173, *p *= 0.4983; Fig. 2*H,K,L*). In the dCA1 (Fig. 3*A,G*), the frequency of sIPSCs was significantly decreased in both 10 and 15 mg/kg MAM-exposed mice (*F*_(2,25) _= 10.15, *p *= 0.0006; Fig. 3*B–D*). Interestingly, this alteration persisted from the adolescent stage to adulthood (*F*_(2,29) _= 8.831, *p *= 0.001; Fig. 3*H–J*). However, the amplitude of sIPSCs did not change in either adolescence (*p *= 0.2767; Fig. 3*B,E,F*) or adulthood (*F*_(2,29) _= 0.79, *p *= 0.4634; Fig. 3*H,K,L*). Similar pathologies were observed in vCA1 (Fig. 4*A,G*), where the frequency of sIPSCs was attenuated in the MAM-exposed mice in both adolescence (*F*_(2,38) _= 5.962, *p *= 0.0056; Fig. 4*B–D*) and adulthood (*F*_(2,42) _= 12.17, *p *< 0.0001; Fig. 4*H–J*), whereas the amplitude was not affected in any condition (adolescence, *F*_(2,38) _= 2.229, *p *= 0.1216; Fig. 4*B,E,F*; adulthood, *p *= 0.489; Fig. 4*H,K,L*]. Altogether, our results suggested that, in MAM-exposed mice, the inhibition toward pyramidal neurons was impaired in mPFC and CA1 during both adolescence and adulthood.

### A mitigation of spike firing of putative interneurons and enhanced putative pyramidal neurons in mPFC and CA1 in vivo

**Figure 5.**
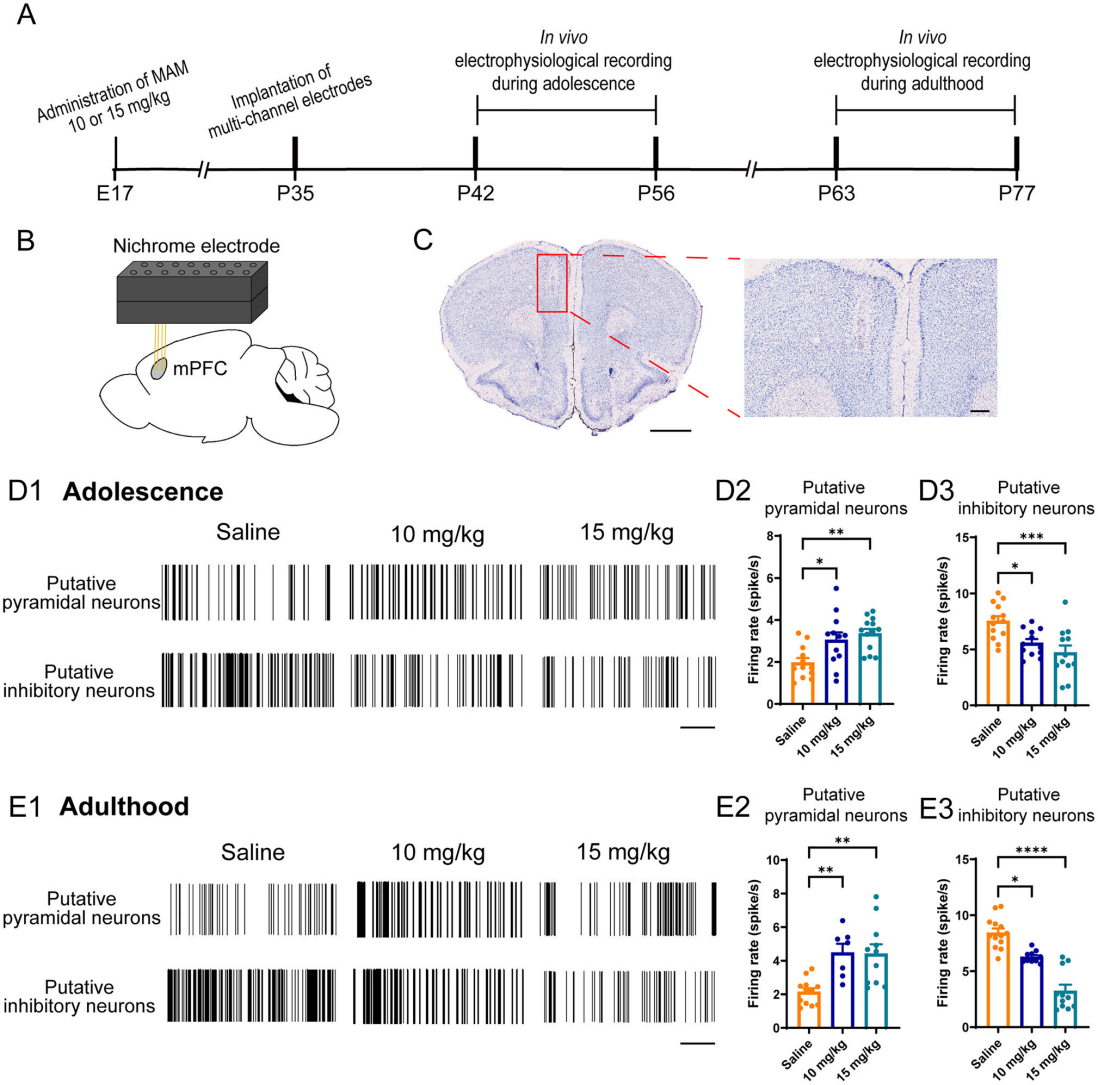
Abnormal spike firing of putative pyramidal neurons and putative inhibitory neurons in mPFC of MAM-exposed mice. ***A***, Schematic diagram of in vivo electrophysiological recordings. ***B***, Schematic representation of the multichannel electrodes implanted in the mPFC. ***C***, Nissl staining plot of electrodes implanted in the mPFC. Scale bars: 1 mm (left), 250 μm (right). ***D*1**, Illustration of mPFC putative pyramidal neurons and putative inhibitory neurons firing spikes during adolescence. Scale bar, 60 s. ***D*2**, ***D*3**, Quantification of mean firing rate of mPFC putative pyramidal neurons (***D*2**) and putative inhibitory neurons (***D*3**). ***E*1**, Illustration of mPFC putative pyramidal neurons and putative inhibitory neurons firing spikes during adulthood. Scale bar, 60 s. ***E*2**, ***E*3**, Quantification of mean firing rate of mPFC putative pyramidal neurons (***E*2**) and putative inhibitory neurons (***E*3**). Each scatter represents a signal unit from 3 to 5 mice per group. Data are mean ± SEM. *p < 0.05, **p < 0.01, ***p < 0.001, ****p < 0.0001. Figure contributions: X.T. performed the experiments and analyzed the data.

**Figure 6.**
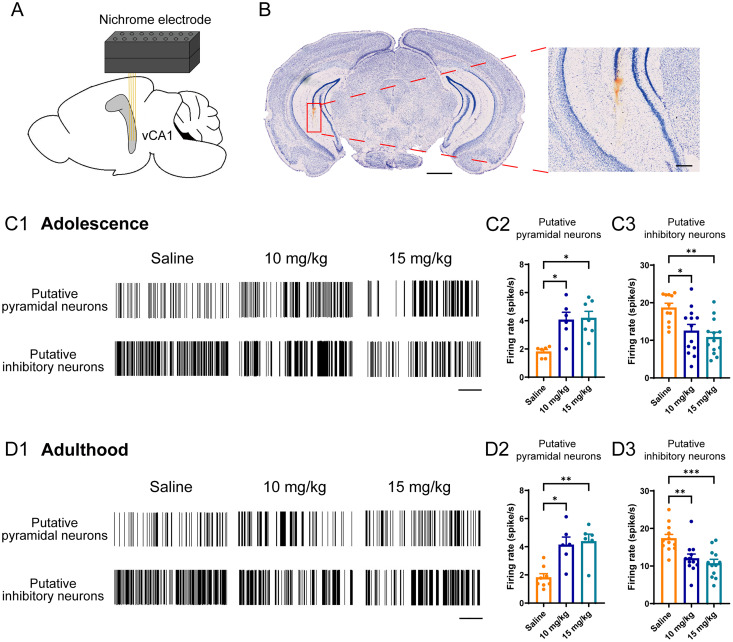
Abnormal spike firing of putative pyramidal neurons and putative inhibitory neurons in vCA1 of MAM-exposed mice. ***A***, Schematic representation of the multichannel electrodes implanted in the vCA1. ***B***, Nissl staining plot of electrodes implanted in the vCA1. Scale bars: 1 mm (left), 250 μm (right). ***C*1**, Illustration of vCA1 putative pyramidal neurons and putative inhibitory neurons firing spikes during adolescence. Scale bar, 60 s. ***C*2**, ***C*3**, Quantification of mean firing rate of vCA1 putative pyramidal neurons (***C*2**) and putative inhibitory neurons (***C*3**). ***D*1**, Illustration of vCA1 putative pyramidal neurons and putative inhibitory neurons firing spikes during adulthood. Scale bar, 60 s. ***D*2**, ***D*3**, Quantification of mean firing rate of vCA1 putative pyramidal neurons (***D*2**) and putative inhibitory neurons (***D*3**). Each scatter represents a signal unit from 3 to 5 mice per group. Data are mean ± SEM. *p < 0.05, **p < 0.01, ***p < 0.001. Figure contributions: X.T. performed the experiments and analyzed the data.

To identify whether the neuronal activity in vivo was affected under MAM exposure, we performed in vivo multichannel recordings in the mPFC (Fig. 5*A–C*) and vCA1 (Fig. 6*A,B*) of both saline-exposed and MAM-exposed mice. Notably, there was evidence that vCA1 was more linked to psychiatric disorders including schizophrenia than dCA1 (O'Reilly et al., 2015), so we chose vCA1 for in vivo electrophysiological recording. We found that in both 10 and 15 mg/kg MAM-exposed mice, the spike firing rate of putative pyramidal neurons in mPFC increased in both adolescence (*F*_(2,34) _= 7.075, *p *= 0.0027; Fig. 5*D*1*,D*2) and adulthood (*F*_(2,27) _= 9.612, *p *= 0.0007; Fig. 5*E*1*,E*2), while the spike firing rate of putative inhibitory neurons decreased in either period (adolescence, *F*_(2,34) _= 8.907, *p *= 0.0008; Fig. 5*D*1*,D*3; adulthood, *p *< 0.0001; Fig. 5*E*1*,E*3). Consistent with the pathology that occurred in the mPFC, the putative pyramidal neuronal activity was significantly increased (adolescence, *p *= 0.0025; Fig. 6*C*1*,C*2; adulthood, *p *= 0.0006; Fig. 6*D*1*,D*2) while the putative inhibitory neuronal activity was impaired (adolescence, *F*_(2,34) _= 7.887, *p *= 0.0025; Fig. 6*C*1*,C*3; adulthood, *F*_(2,33) _= 10.33, *p *= 0.0003; Fig. 6*D*1,*D*3) in vCA1 of the MAM-exposed mice, compared with that in the saline exposure mice. In conclusion, there was a decrease of spike firing in putative inhibitory neurons along with an increase of putative pyramidal neurons in vivo both in the mPFC and vCA1 of the MAM-exposed mice during either adolescence or adulthood. Moreover, since the previous experiments in this study indicated that the pathology between dCA1 and vCA1 in MAM-exposed mice was comparable, it was speculated that the neuronal activity in dCA1 in vivo of MAM-exposed mice might be similar to those in vCA1.

## Discussion

In the study, we found that 10 or 15 mg/kg MAM intraperitoneal injection into the pregnant mice caused comparable pathological changes in their offspring, including a loss of PV neurons, a lower frequency of sIPSCs in pyramidal neurons, increased discharges in vivo in putative pyramidal neurons, and a decrease in putative inhibitory neurons at mPFC and hippocampus during either adolescence or adulthood. These results suggest that enhancing the function of PV neurons in the mPFC and hippocampus in individuals susceptible to schizophrenia or schizophrenia may provide a strategy of intervention in schizophrenia. Consistent with this insight, a previous study indicated that enhancement of PV neuron function by a novel α5GABA(A)R-positive allosteric modulator was effective in restoring hippocampal output and, in turn, normal activity of dopamine neurons (Gill et al., 2011).

The study has explored the pathophysiological alterations in low doses of MAM-exposed mice during adolescence and adulthood. A large number of characteristics, such as excitation–inhibition imbalance (Yizhar et al., 2011), a diminished excitatory synaptic transmission (Zhang et al., 2021), and an enhanced number of spontaneous firing neurons in the ventral tegmental area (Zhu and Grace, 2021), have been suggested to involve in the pathogenesis of schizophrenia. In this study, there was an obvious PV loss in the MAM-exposed mice. Considering that the PV neurons project to the soma and axon initial segments of pyramidal neurons and thus regulate the neuronal activity (Markram et al., 2004), it was suggested that the loss of PV could contribute to reduced inhibitory output to pyramidal neurons and disturbed neuronal activity in vivo manifesting a significant decrease of inhibitory neuronal discharges and an obvious increase of pyramidal neuronal discharges. Furthermore, the frequency of sIPSCs in pyramidal neurons was reduced in both mPFC and CA1 of MAM-exposed mice. Our finding is supported by a study that hypoexcitability in PV neurons of the ACC resulted in an attenuation in inhibitory output to pyramidal neurons in MAM-exposed mice (Zhang et al., 2023). Moreover, the previous study indicated that the loss of PV neurons was associated with diminished oscillatory activity in MAM-exposed rats (Lodge et al., 2009). Intriguingly, PV neurons generated from embryonic stem cell lines, transplanting into the ventral hippocampus of MAM-exposed rats, reduced hippocampal hyperactivity and alleviated positive, negative, and cognitive symptoms (Donegan et al., 2017). These results provided the rationale for MAM exposure during pregnancy inducing the schizophrenia-like phenotypes in the offspring. Altogether, we speculated that the loss of PV neurons may contribute to the impaired inhibitory function, as well as the disturbance of neuronal activity. It should be noted that other types of inhibitory neurons, like cholecystokinin-, vasoactive intestinal polypeptide-, and somatostatin-positive neurons, were also decreased in the hippocampus in MAM-exposed rats (Zoli et al., 1990; Donegan et al., 2017).

Although the majority of cases of schizophrenia have onset in late adolescence or early adulthood, a minority of patients have onset in early adolescence or even childhood (Häfner et al., 1989; Häfner and an der Heiden, 1997; Solmi et al., 2022). Adolescence is a critical period for the structural and functional rewiring of the brain (Lebel et al., 2008) and is also associated with an increased vulnerability to schizophrenia onset in adulthood (Patel et al., 2021). As schizophrenia is increasingly recognized as a neurodevelopmental disorder, early intervention leads to better outcomes than delayed treatment (Salazar de Pablo et al., 2020; Arango et al., 2021; Catalan et al., 2021). Our results demonstrated that abnormal pathological changes have been observed in mPFC and CA1 in the offspring of MAM-exposed dam during adolescence, including the loss of PV neurons, decreased frequency of sIPSCs in vitro, and disturbed neuronal activity in vivo, which sustained to adulthood. In this case, early intervention can be considered to be carried out in schizophrenia or susceptible populations, whose may exhibit attenuated inhibitory function in the brain, and thus the prognosis may be better.

Twenty to twenty six milligram per kilogram of MAM is widely used in rodents to establish the schizophrenia-like model (Moore et al., 2006; Lodge, 2013; Gill et al., 2018; Zhang et al., 2023; Liang et al., 2024), and there are fewer reports in mice than that in rats. Moreover, the dosage of MAM used in mice is variable. In this case, we investigated two dosages of MAM (10 and 15 mg/kg) to analyze the underlying pathologies of mice. It was shown that schizophrenia-like behavior phenotypes and electrophysiological-related changes were found in mice at both 10 and 15 mg/kg. As mentioned in the results, 20 mg/kg MAM would result in an extremely low survival of the offspring of mice in the condition, which was different from other reports (Gill et al., 2014, 2018). Consistent to our result, a study of mice given different concentrations of MAM (2.5, 5, 7.5, 10, and 25 mg/kg) showed that MAM injection at concentrations over 10 mg/kg caused spontaneous abortion in mice and, surprisingly, 7.5 mg/kg of MAM was sufficient to induce pathological changes and schizophrenia-like behaviors in their offspring (Huo et al., 2018). Accordingly, it's a limitation of this study that no more doses of MAM have been conducted to validate these pathologies.

There are notable gender differences in the pattern of the disorder. Men tend to experience schizophrenia-related symptoms earlier in life and may have a more severe course of illness (Ferrara et al., 2024), while women often have a later onset and may experience better social functioning, possibly due to the protective effects of estrogen (Seeman, 2000; Culbert et al., 2022). Considering the differences in the progression of disorder by sex and the fact that our study of mice started from puberty, only male mice were included. Nonetheless, it is important to further investigate the effects of MAM on different sex.

In conclusion, we reported an attenuated expression of PV neurons in the mPFC and hippocampus, which may be responsible for the impaired inhibition of the pyramidal neurons, and a hyperactivity of pyramidal neurons in vivo. These might be an intrinsic mechanism of schizophrenia onset at all stages of life, which focus on upstream pathophysiology and aim to alleviate positive symptoms and cognitive and negative symptoms.

## Synthesis

Reviewing Editor: Jibran Khokhar, Western University Department of Anatomy and Cell Biology Decisions are customarily a result of the Reviewing Editor and the peer reviewers coming together and discussing their recommendations until a consensus is reached. When revisions are invited, a fact-based synthesis statement explaining their decision and outlining what is needed to prepare a revision will be listed below. The following reviewer(s) agreed to reveal their identity: Anthony Grace, Melissa Perreault.

Summary:

The manuscript investigates inhibitory function in the mPFC and hippocampus using the MAM mouse model of schizophrenia, translating findings from the well-established rat model. The study examines the impact of prenatal MAM exposure on excitatory/inhibitory balance across development, focusing on PV neuron function. While the manuscript is generally well-done and presents findings clearly, there are significant concerns about the validation of the model as a schizophrenia model and the translational relevance of the findings. Issues related to dosing justification, inclusion of both sexes, and the description of experimental results require attention.

Major Concerns:

1. MAM Dose Justification: There is no citation or adequate justification for the MAM doses used, which differ from established literature, such as Chalkiadaki et al. (2019) and Jiang et al. The relevance of these doses to schizophrenia models is questionable.

2. Schizophrenia Model Validation: The manuscript needs a more thorough discussion on the validation of the MAM model as a schizophrenia model. As it stands, the work may be better framed as a study on the impact of low-dose MAM on inhibitory/excitatory function, not schizophrenia.

3. Exclusion of Female Animals: Female mice were excluded despite significant sex differences in schizophrenia. Both sexes should be included, or at least their exclusion needs to be justified or discussed as a limitation.

4. Insufficient Description of Results: Figures 2-4 are inadequately described. A clearer explanation of what the curves represent is needed, particularly if the inter-event interval represents action potential intervals rather than frequency.

5. Reevaluation of Schizophrenia References: The manuscript's references to schizophrenia need reconsideration, particularly in light of dosing concerns and behavioral model validation.

Minor Concerns:

1. Introduction - Antipsychotic Drugs: First- and second-generation antipsychotics do not effectively treat negative or cognitive symptoms of schizophrenia, and this should be clarified.

2. Terminology: The term "hyperactive hippocampus" needs clarification, and terms like "causes" should be replaced with less definitive phrasing like "plays an important role."

3. Title and Abstract: Both need revision to be more precise, particularly in describing the impaired inhibition and inhibitory function.

4. In Situ vs. In Vitro: The terminology for slice experiments should be "in situ," not "in vitro."

5. Methods, Figures and Formatting**: Figures were out of order in the merged file, and the figure number should be clearly included. Housing conditions should also be clarified, including details on enrichment.

6. Spelling/Grammar: There are minor spelling and grammatical issues throughout the text that need to be corrected.

7. Line 261 - a study by Gill and Grace showed that enhancing PV neuron efficacy via a GABA A alpha 5 PAM was effective in normalizing hippocampal activity and restoring normal dopamine neuron function
