# Supplementary figures and images for: Prenatal Exposure to MAM Impairs mPFC and Hippocampal Inhibitory Function in Mice during Adolescence and Adulthood
Source: eNeuro. 2024 Nov 14;11(11):ENEURO.0362-24.2024. doi: 10.1523/ENEURO.0362-24.2024 (PMC11625879; doi:10.1523/ENEURO.0362-24.2024)

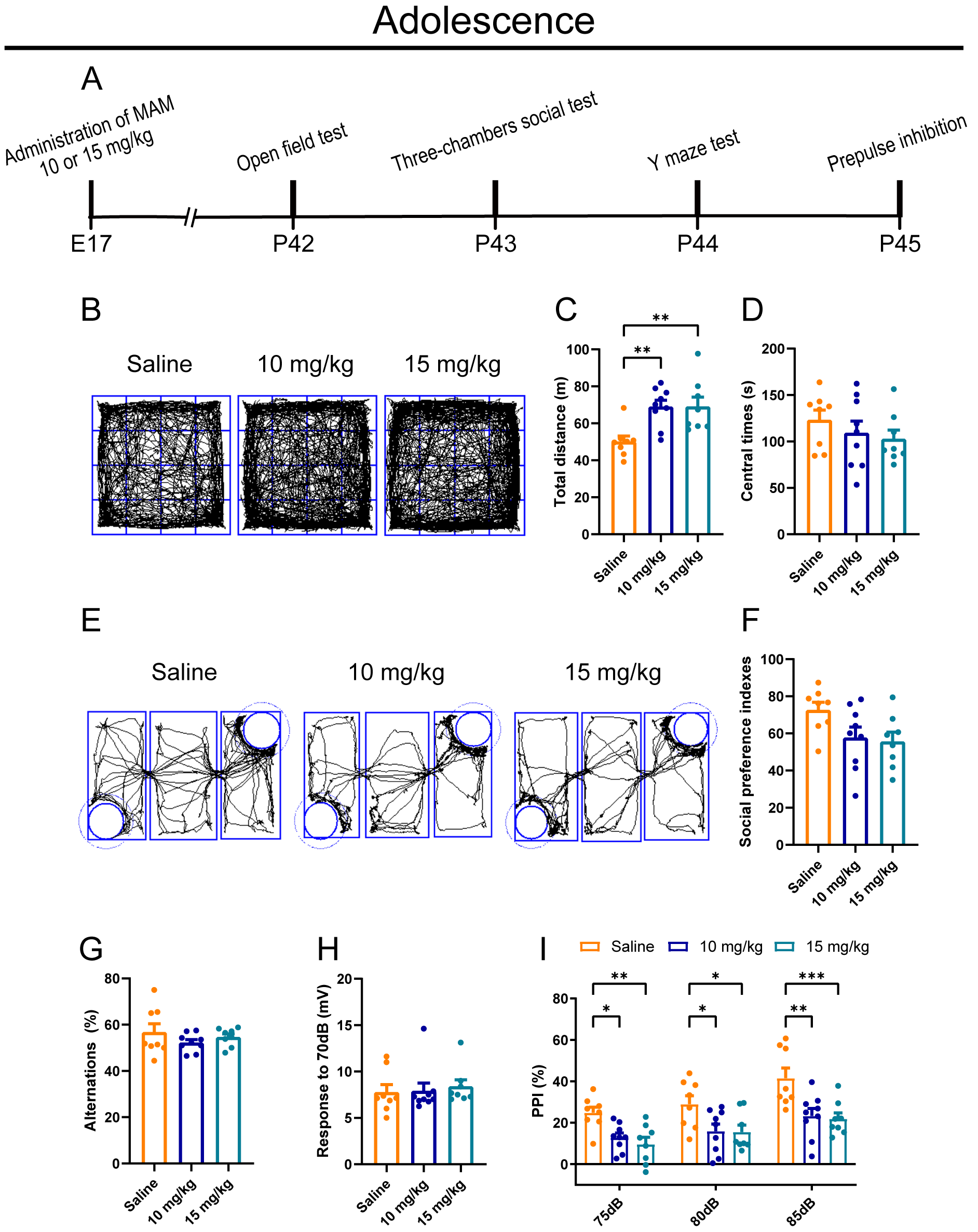

Supplement: Figure 1-1 — 10 mg/kg and 15 mg/kg MAM-exposed mice exhibited schizophrenia-like behaviors during adolescence. A, Schematic representation of Saline- and MAM (10 mg/kg and 15 mg/kg)- exposed mice undergoing a series of schizophrenia-related behavioral tests during adolescence. B-D, MAM exposure induced locomotor hyperactivity in the open field test. Representative activity tracking (B), total distance (C) and time in center (D) [(C) F(2,22) = 7.616, p = 0.0031; (D) F(2,22) = 0.852, p = 0.4400]. E, F, No changes in social activity were observed. Representative activity tracking (E) and social preference indexes (F) in three-chambers social test [F(2,22) = 3.207, p = 0.0599]. G, No changes in alternation (%) in Y maze test [F(2,22) = 0.953, p = 0.4011]. H, I, MAM exposure induced an impaired PPI. Quantification of response to 70 dB (E) and percentage of PPI (F) in prepulse inhibition test [(E) p = 0.2761; (F) Two-way ANOVA, interaction: F(4, 66) = 0.323, p = 0.8619; main effect of decibel: F(2, 66) = 11.230, p < 0.0001; main effect of group: F(2, 66) = 19.120, p < 0.0001]. N = 8, 9, 8 mice per group. Data are mean ± SEM. *p < 0.05, **p < 0.01, ***p < 0.001. Figure Contributions: Zhiyin He and Keni Huang performed the experiments and analyzed the data. Download Figure 1-1, TIF file. [file eneuro-11-ENEURO.0362-24.2024-s002.tif]

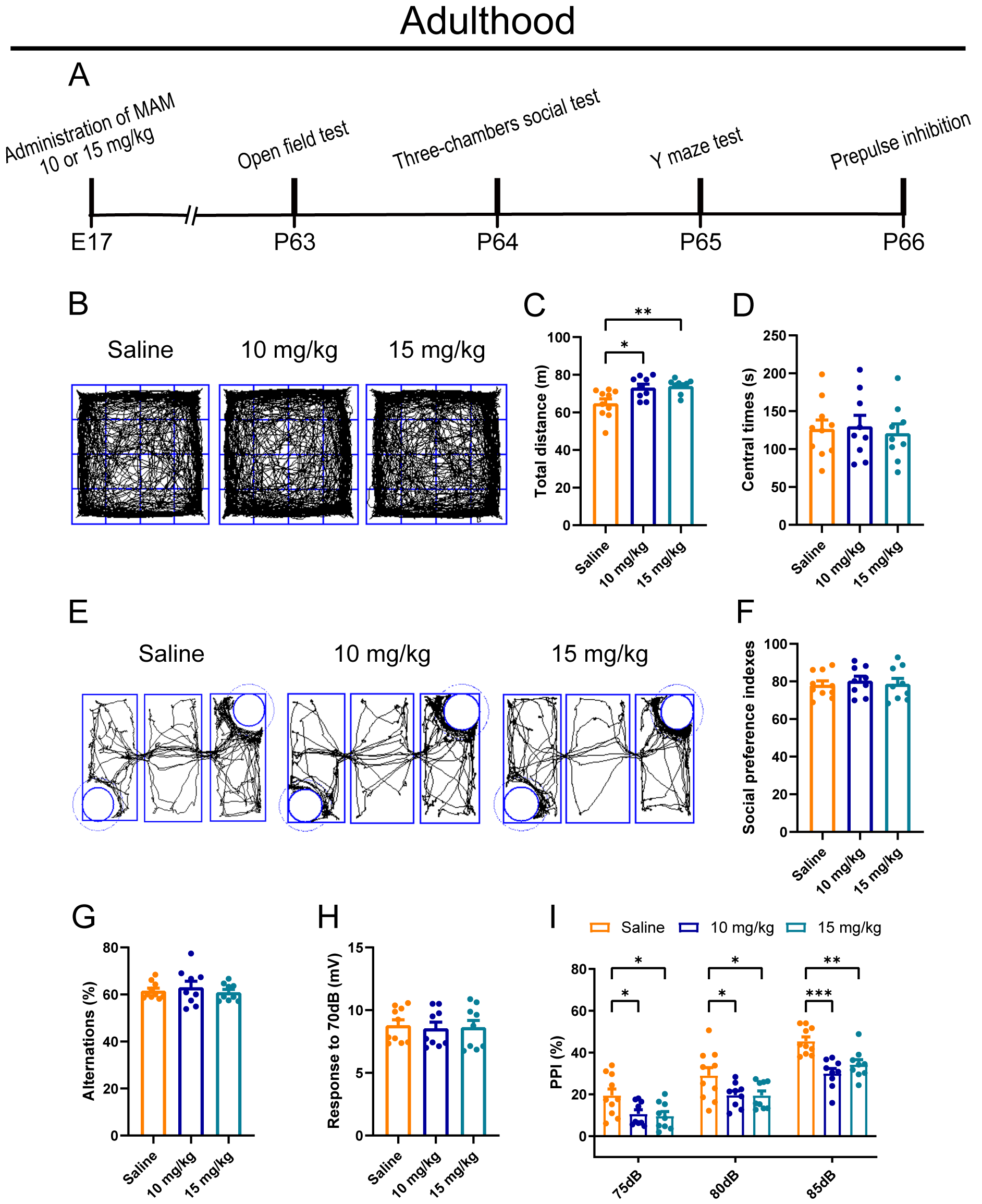

Supplement: Figure 1-2 — 10 mg/kg and 15 mg/kg MAM-exposed mice exhibited schizophrenia-like behaviors during adulthood. A, Schematic representation of Saline- and MAM (10 mg/kg and 15 mg/kg)- exposed mice undergoing a series of schizophrenia-related behavioral tests during adolescence. B-D, MAM exposure induced locomotor hyperactivity in the open field test. Representative activity tracking (B), total distance (C) and time in center (D) [(C) F(2,25) = 6.897, p = 0.0041; (D) F(2,25) = 0.122, p = 0.8858]. E, F, No changes in social activity were observed. Representative activity tracking (E) and social preference indexes (F) in three-chambers social test [F(2,25) = 0.200, p = 0.8201]. G, No changes in alternation (%) in Y maze test [F(2,25) = 0.394, p = 0.6787]. H, I, MAM exposure induced an impaired PPI. Quantification of response to 70 dB (E) and percentage of PPI (F) in prepulse inhibition test [(E) p = 0.7745; (F) Two-way ANOVA, interaction: F(4, 75) = 0.575, p = 0.6820; main effect of decibel: F(2, 75) = 64.010, p < 0.0001; main effect of group: F(2, 75) = 18.370, p < 0.0001]. N = 10, 9, 9 mice per group. Data are mean ± SEM. *p < 0.05, **p < 0.01, ***p < 0.001. Figure Contributions: Zhiyin He and Qian He performed the experiments and analyzed the data. Download Figure 1-2, TIF file. [file eneuro-11-ENEURO.0362-24.2024-s003.tif]
